# Supplementary material for: Trehalose Modulates Autophagy Process to Counteract Gliadin Cytotoxicity in an In Vitro Celiac Disease Model
Source: Cells. 2019 Apr 12;8(4):348. doi: 10.3390/cells8040348 (PMC6523171; doi:10.3390/cells8040348)
Supplement: Supplementary file 1 [file cells-08-00348-s001.pdf]

## Supplementary Materials

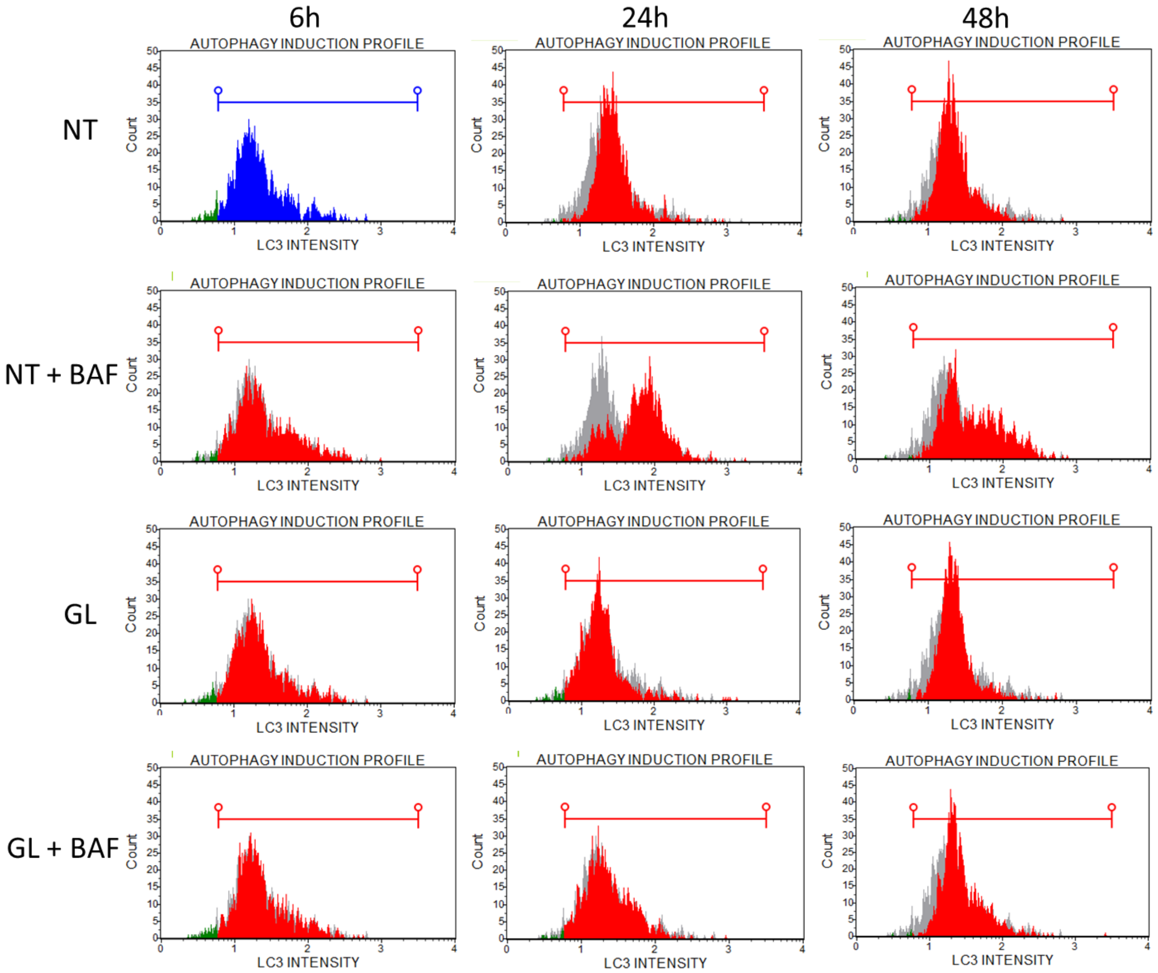


**Supplementary Figure 1:** Cytofluorimetric plots representing LC3-II expression in Caco-2 cells incubated with digested gliadin (1 µg/µl) at different time intervals (i.e. 6-24-48 hours p.t.). The plot of the non-treated sample (NT) in represented in grey whereas in red are reported the plots of the different treatments. Bafilomycin A1 (10 nM) was used to study the autophagy flux. The analysis was performed with Muse® Cell Analyzer.

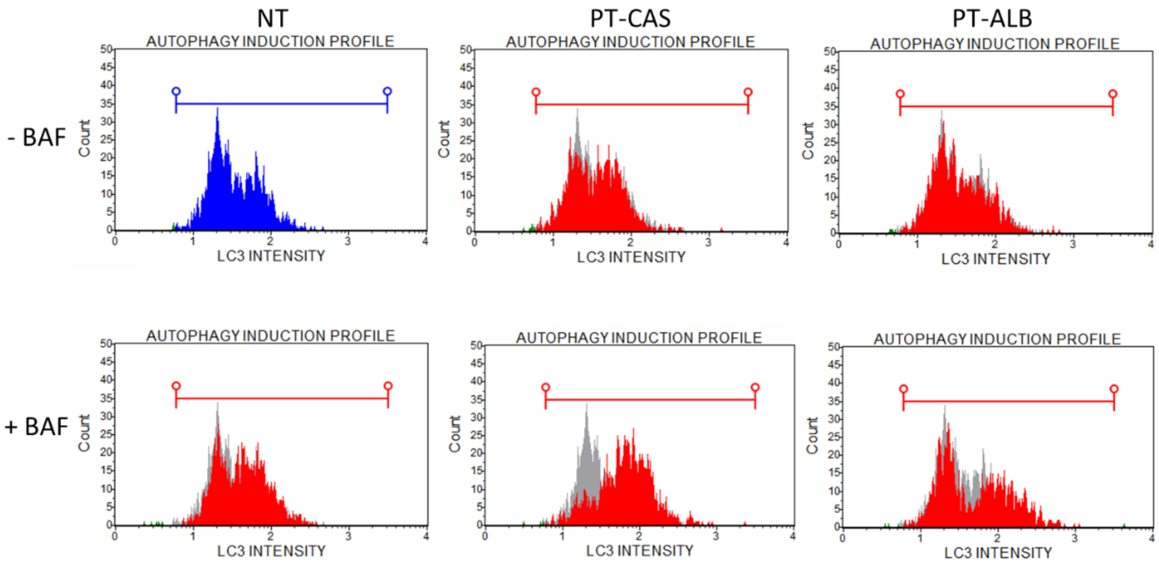


**Supplementary figure 2:** Cytofluorimetric plots representing LC3-II expression in Caco-2 cells incubated with digested casein or albumin (1 µg/µl) at 24 hours p.t. The plot of the non-treated sample (NT) is represented in grey whereas in red are reported the plots of the different treatments. Bafilomycin A1 (10 nM) was used to study the autophagy flux. The analysis was performed with Muse® Cell Analyzer.

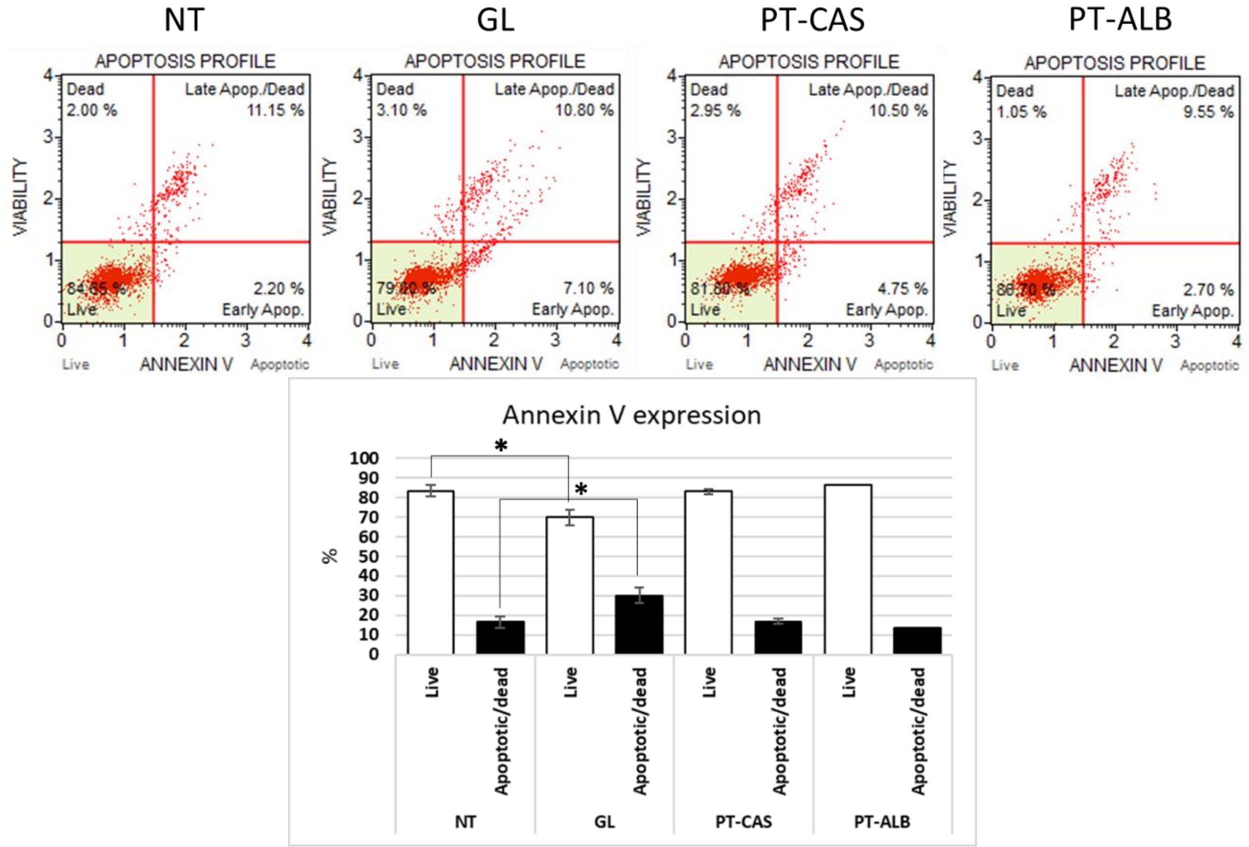


**Supplementary figure 3:** Cytofluorimetric plots and derived graph showing apoptotic levels of Caco-2 cells treated with digested gliadin, casein or albumin (1 µg/µl) at 24 hours p.t. The analysis was performed with Muse® Cell Analyzer. Annexin V densitometric analysis was performed by Muse® Cell Analyzer (Merck) at 24 hours p.t. SE bars are reported. Asterisks indicates p<0.05, Anova One-Way, compared with NT sample. The experiments were performed in triplicates. Cytofluorimetric plots are reported in Supplementary Figure 3.

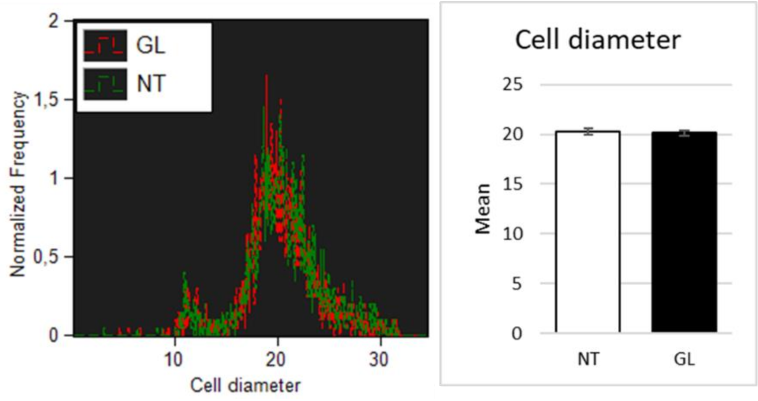


**Supplementary figure 4:** Morphological analysis of Caco-2 cells treated with PT-gliadin (1 µg/µl). Cell were analyzed 24 hours p.t. and treate cells were compared with non-treated (NT) ones. Event collected: 2000. The analysis was performed with Amnis ImageStream X Mark II (Merck).

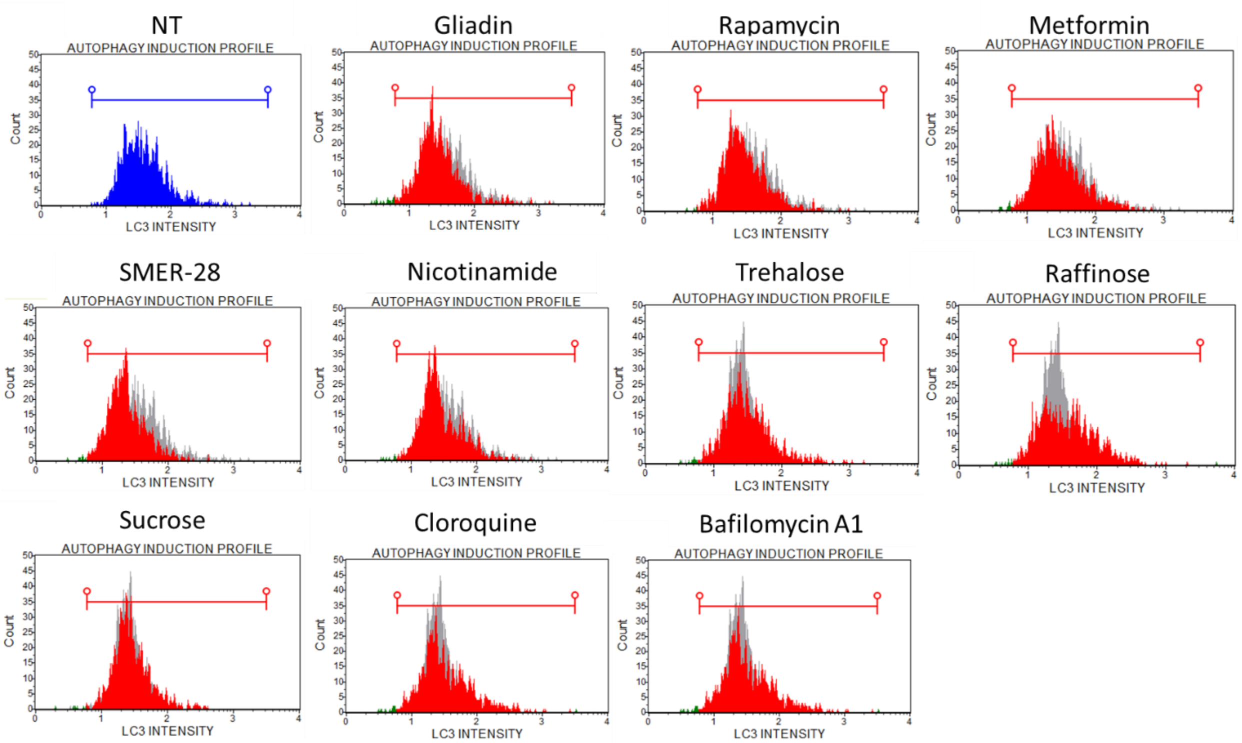


**Supplementary Figure 5:** Cytofluorimetric plots showing LC3-II expression in Caco-2 cells treated with different autophagy inducers: rapamycin (5 µM), metformin (5 mM), SMER-28 (50 µM), nicotinamide (5 mM), trehalose (100 mM), raffinose (100 mM), sucrose (100 mM), cloroquine (25 µM) and bafilomycin A1 (10 nM). The analysis was performed with Muse® Cell Analyzer.

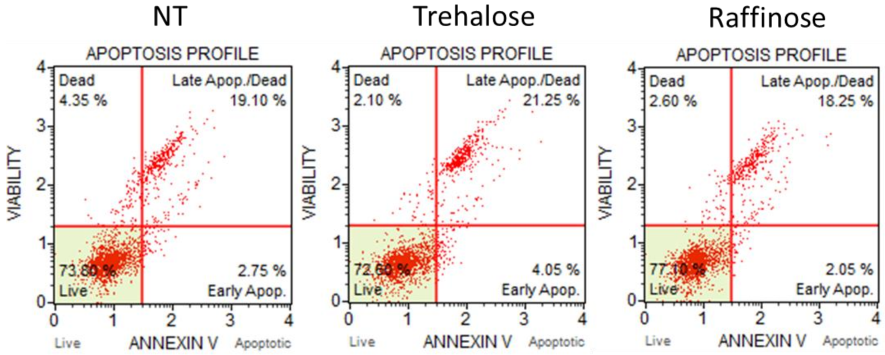


**Supplementary figure 6:** Cytofluorimetric plots showing apoptotic levels of Caco-2 cells treated with trehalose and raffinose (100 mM) at 24 hours p.t. The analysis was performed with Muse® Cell Analyzer.

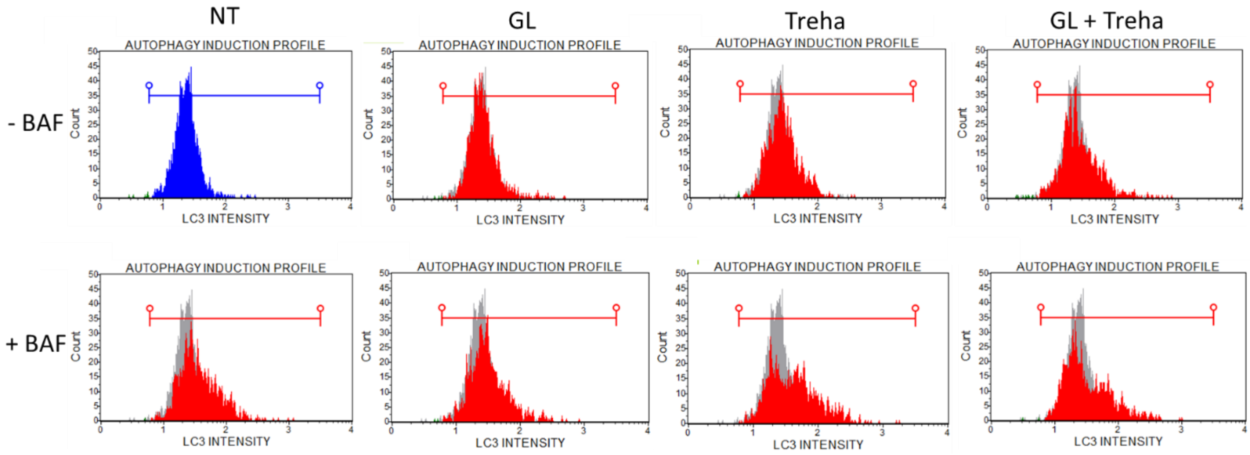


**Supplementary Figure 7:** Cytofluorimetric plots representing LC3-II expression levels in Caco-2 cells treated with PT gliadin (1 µg/µl), trehalose (100 mM) or the combination of both. Bafilomycin A1 (10 nM) was used to investigate autophagy flux. The analysis was performed with Muse® Cell Analyzer.

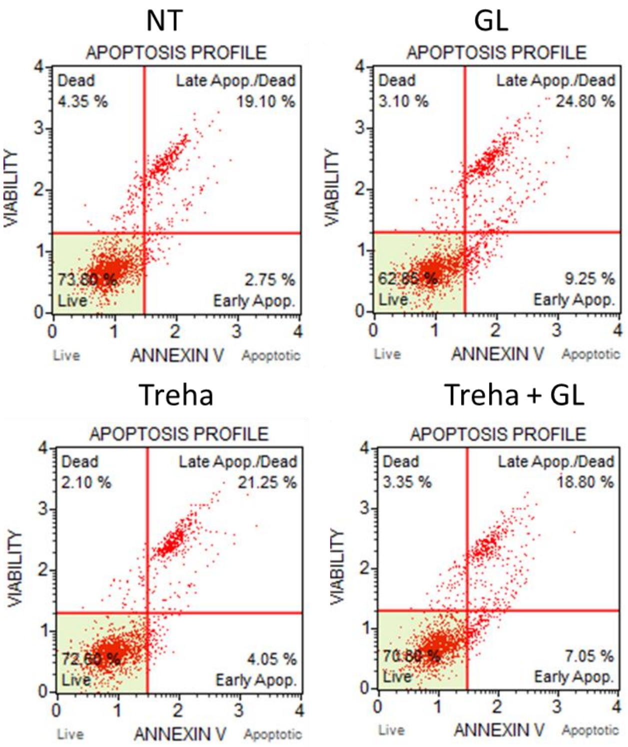


**Supplementary Figure 8:** Cytofluorimetric plots showing apoptotic levels of Caco-2 cells treated with digested gliadin (1 µg/µl), trehalose (100 mM) or the combination of both at 24 hours p.t. The analysis was performed with Muse® Cell Analyzer.

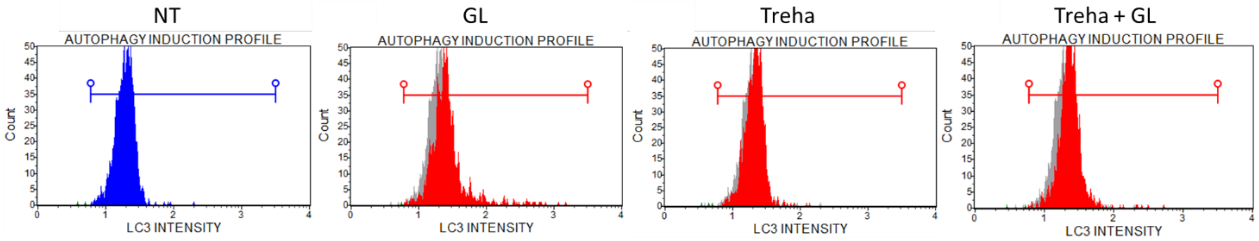


**Supplementary Figure 9:** Cytofluorimetric analysis of the intracellular amount of GLIA-555 (1 µg/µl) in presence/absence of trehalose (100 mM) after 24 hours p.t. The analysis was performed with Muse® Cell Analyzer.

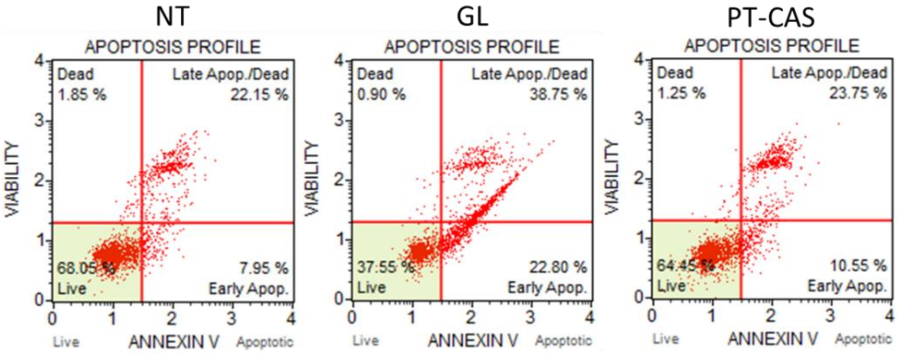


**Supplementary figure 10:** Cytofluorimetric plots showing apoptotic levels of HT-29 cells treated with digested gliadin and casein (1 µg/µl) at 24 hours p.t. The analysis was performed with Muse® Cell Analyzer.

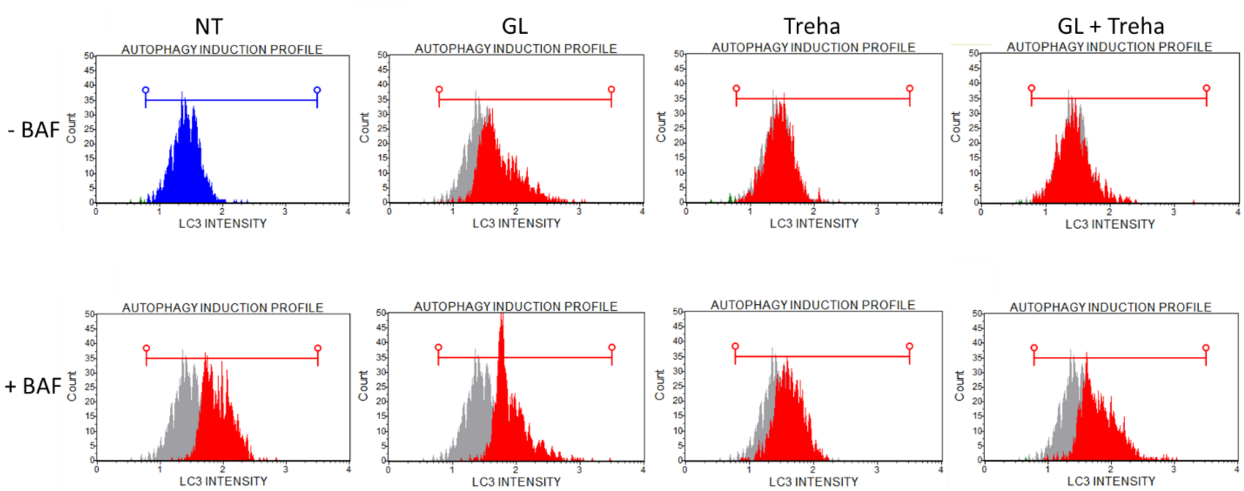


**Supplementary figure 11:** Cytofluorimetric plots representing LC3-II expression levels in HT-29 cells treated with PT gliadin (1 µg/µl), trehalose (100 mM) or the combination of both. Bafilomycin A1 (10 nM) was used to investigate autophagy flux. The analysis was performed with Muse® Cell Analyzer.
